# Série temporal da produção odontológica no Sistema Único de Saúde, Brasil, 2008-2018

**DOI:** 10.1590/S1679-49742022000100007

**Published:** 2022-03-21

**Authors:** Georgia Costa de Araújo Souza, Sandro Alves Mourão, Gustavo Barbalho Guedes Emiliano

**Affiliations:** 1 Universidade Federal do Rio Grande do Norte, Departamento de Odontologia, Natal, RN, Brasil Universidade Federal do Rio Grande do Norte Universidade Federal do Rio Grande do Norte Departamento de Odontologia Natal RN Brazil; 2 Universidade do Estado do Rio Grande do Norte, Departamento de Odontologia, Caicó, RN, Brasil Universidade do Estado do Rio Grande do Norte Universidade do Estado do Rio Grande do Norte Departamento de Odontologia Caicó RN Brazil

**Keywords:** Saúde Bucal, Sistemas de Informação, Vigilância em Saúde Pública, Assistência Ambulatorial, Estudos de Séries Temporais., Salud Bucal, Sistemas de Información, Vigilancia en Salud Pública, Atención Ambulatoria, Estudios de Series Temporales., Oral Health, Information Systems, Public Health Surveillance, Outpatient Care, Time Series Studies.

## Abstract

**Objetivo:**

Analisar a realização de procedimentos odontológicos pelo Sistema Único de Saúde (SUS), no Brasil e suas regiões geopolíticas, entre 2008 e 2018.

**Métodos:**

Estudo de série temporal, com dados do Sistema de Informações Ambulatoriais dos SUS. Foram calculadas taxas anuais de procedimentos odontológicos (por 100 mil habitantes), globais e por categorias de procedimentos e regiões. Utilizou-se a regressão de Prais-Winsten para analisar as tendências temporais e foram calculadas variações percentuais anuais (VPA).

**Resultados:**

Tendências decrescentes foram encontradas para o Brasil, em ações coletivas (VPA= -13,5%; IC_95%_ -21,1;-5,2), ações preventivas individuais (VPA= -6,2%; - IC_95%_ -7,7;-4,8), restaurações (VPA= -7,3%; IC_95%_ -10,5;-3,9) e exodontias (VPA= -6,9; IC_95%_ -10,5;-3,1). Endodontia e periodontia apresentaram tendências estacionárias para a maioria das regiões e o Brasil. Procedimentos protéticos apresentaram tendência ascendente em todas as regiões e no Brasil (VPA= 16,9%; IC_95%_ 9,1;25,2).

**Conclusão:**

A produção odontológica no SUS apresentou decréscimo no período 2008-2018; à exceção de procedimentos protéticos, cuja produção aumentou.

## Abstract (es)

**Objetivo:**

Analizar el desempeño de procedimientos dentales en el Sistema Único de Salud (SUS) en Brasil y regiones, 2008 - 2018.

**Métodos:**

Estudio de series temporales con datos del Sistema de Información Ambulatoria. Se calcularon tasas anuales de procedimientos dentales por 100 mil habitantes, globales y según categorías de procedimientos y regiones brasileñas de 2008 a 2018. Se utilizó la regresión de Prais-Winsten para analizar tendencias temporales y se calcularon los cambios porcentuales anuales.

**Resultados:**

Hubo tendencia decreciente en Brasil para acciones colectivas -13,5%; IC_95%_ -21,1;-5,2), acciones preventivas individuales (-6,2%; IC_95%_ -7,7;-4,8), restauraciones (-7,3%; IC_95%_ -10,5;-3,9) y extracciones (-6,9; IC_95%_ -10,5;-3,1%). Endodoncia y periodoncia mostraron tendencias estacionarias para la mayoría de las regiones y Brasil. Procedimientos protésicos mostraron tendencia ascendente en todas las regiones y Brasil (16,9%; IC_95%_ 9,1;25,2).

**Conclusión:**

La producción dental en el SUS disminuyó entre 2008 y 2018, a excepción de los procedimientos protésicos cuya producción aumentó.

## Abstract (en)

**Objective:**

To analyze dental procedures provided by the Brazilian National Health System (SUS) in Brazil and its macro-regions, between 2008 and 2018.

**Methods:**

This was a time series study using data from SUS Outpatient Information System. Annual and overall rates of dental procedures (per 100,000 inhabitants), according to the categories of dental procedures and regions were calculated. Prais-Winsten regression was used to analyze time trends, while annual percentage change (APC) was calculated.

**Results:**

Decreasing trends were found in Brazil, in collective measures (APC= -13.5%; 95%CI -21.1;-5.2), individual preventive measures (APC= -6.2%; 95%CI -7.7;-4.8), dental restoration (APC= -7.3%; 95%CI -10.5;-3.9) and tooth extraction procedures (APC= -6.9; 95%CI -10,5;-3,1). Endodontics and periodontics showed stationary trend in most regions and Brazil. Prosthetic procedures showed an upward trend in all regions and Brazil (APC= 16.9%; 95%CI 9.1;25.2).

**Conclusion:**

Dental procedures in the SUS decreased between 2008-2018; with the exception of prosthetic procedures, which showed a rising trend.

## INTRODUÇÃO

**Table:** 

Contribuições do estudo	
Principais resultados	A produção odontológica no SUS apresentou tendência de declínio entre 2008 e 2018, à exceção de procedimentos protéticos, cuja tendência foi ascendente.
Implicações para os serviços	O estudo deve auxiliar os profissionais de saúde bucal e os gestores na identificação da redução dos procedimentos ofertados, estimulando o acompanhamento e monitoramento da produção odontológica em nível local.
Perspectivas	Busca por soluções macro e micro para suprir demandas dos serviços odontológicos, diante da tendência de declínio desta produção. Futuros estudos devem analisar produção, financiamento, recursos humanos e demanda dos serviços odontológicos no SUS.

A inserção de equipes de Saúde Bucal na Estratégia Saúde da Família (ESF) proporcionou a reorganização dos serviços de saúde bucal na atenção básica e maior oferta de procedimentos odontológicos pelo Sistema Único de Saúde (SUS).^1^ O aumento do acesso à saúde bucal foi observado na parcela da população mais vulnerável, apesar das dificuldades enfrentadas pelo sistema, como a fragmentação das políticas e programas de saúde, da qualificação da gestão e do controle social, além da dificuldade para organizar uma rede regionalizada e hierarquizada de ações e serviços de saúde, comprometendo a universalidade e integralidade do SUS.^2^ Com o estabelecimento das diretrizes da Política Nacional de Saúde Bucal (PNSB) em 2004, importantes avanços foram observados na atenção à saúde bucal, enquanto novos desafios surgiram.^3^

Entre os avanços alcançados, são dignos de nota o maior incentivo às ações programáticas de fluoretação das águas de abastecimento público, a estruturação de uma rede de referência e contrarreferência com os Centros de Especialidades Odontológicas (CEOs) e os Laboratórios Regionais de Prótese Dentária (LRPDs), e um crescimento de 390% no número de equipes de Saúde Bucal na ESF.^4^

Adicionalmente, a ampliação da atenção secundária à saúde, impulsionada pela PNSB, incorporou a oferta de procedimentos de maior complexidade, como terapias protéticas, endodônticas, ortodônticas, apoio diagnóstico (radiologia e biopsias) e outros, reorganizando a rede de serviços de saúde bucal.^4^

A adoção das medidas propostas pela PNSB, contudo, não ocorreu de maneira uniforme em todo o país.^5^ Há, portanto, necessidade de pensar a aplicação das diretrizes do PNSB locorregionalmente, articulada às demais políticas públicas, de forma a propiciar a execução de ações intersetoriais, maior acesso às ações e serviços de saúde de forma integral e a superação das desigualdades sociais.^6^^)^ Compete à epidemiologia fornecer informações sobre o perfil de saúde bucal da população e a organização dos serviços, auxiliando no planejamento em saúde.^7^

Dados do último levantamento nacional de saúde bucal, o SB Brasil 2010, mostraram que a maioria da população brasileira nas idades de 12 (58,1%) e dos 15 aos 19 anos (46,3%) procurou o sistema público para uso de serviços odontológicos, sendo os motivos mais citados para a última consulta a realização de tratamento (33,1% e 37,3%, respectivamente) e revisão ou prevenção (38,3% e 36,2%, respectivamente). Quanto aos adultos e idosos, a maioria realizou a última consulta odontológica no serviço particular (49,1% e 59,8%, respectivamente), sendo os motivos mais citados por esses segmentos (i) a necessidade de tratamento (44,6%) e a prevenção (21,4%) entre os adultos, e (ii) o tratamento (36,8%) e extração (26,9%) entre os idosos.^8^ Estes dados reafirmam características da atenção à saúde bucal, centralizada em práticas clínicas individuais e tecnicistas, inclusive na ESF, justificadas pela falta de estratégias integrais robustas e coordenadas de enfrentamento da grande demanda reprimida.^9^ Considera-se que o conhecimento sobre o perfil de procedimentos ofertados pode contribuir para a análise das características da assistência odontológica. Ademais, a compreensão da tendência temporal da produção odontológica possibilita a identificação de mudanças ocorridas na assistência à saúde bucal, ao longo dos anos.^10^

Existem avaliações da evolução da assistência odontológica nos âmbitos local e regional,^10^^)-(^^12^ mas são poucas as pesquisas em nível nacional^13^^),(^^14^ que investigaram desde o período anterior à inclusão das equipes de Saúde Bucal na Saúde da Família, passando pelos investimentos relacionados à PNSB a partir de 2004, e demonstraram a ascensão nos procedimentos odontológicos no Brasil. A depender do intervalo de tempo e do contexto analisados, os resultados desses estudos podem diferir segundo o perfil da população e a orientação das políticas públicas. O objetivo deste estudo foi analisar a realização de procedimentos odontológicos pelo SUS, no Brasil e em suas regiões geopolíticas, no período entre 2008 e 2018.

## MÉTODOS

Trata-se de um estudo ecológico de série temporal. As unidades de análise foram o Brasil e suas cinco regiões geopolíticas: Norte, Nordeste, Sudeste, Sul e Centro-Oeste. O período investigado compreendeu os anos de 2008 a 2018. O início desse período coincide com a mudança no Sistema de Informações Ambulatoriais do SUS (SIA/SUS), quando houve atualização nas nomenclaturas dos procedimentos e seus respectivos códigos na tabela do SUS.^15^ Neste estudo, foram incluídos os procedimentos odontológicos ambulatoriais da tabela do SUS.

As variáveis do estudo foram as categorias de procedimentos odontológicos, cujas definições e demais informações se encontram no Quadro 1: ações coletivas; ações preventivas individuais; procedimentos restauradores; procedimentos endodônticos; procedimentos exodônticos; procedimentos periodontais; procedimentos protéticos; além de população, anos e dimensões geográficas do Brasil e suas grandes regiões.

**Quadro 1:** - Categorias de procedimentos odontológicos analisados e respectivos códigos no Sistema de Informações Ambulatoriais do Sistema Único de Saúde (SIA/SUS)

Ações coletivas	Atividade educativa/orientação em grupo na atenção básica (0101010010); Ação coletiva de aplicação tópica de flúor gel (0101020015); Ação coletiva de bochecho fluorado (0101020023); Ação coletiva de escovação dental supervisionada (0101020031); Ação coletiva de exame bucal com finalidade epidemiológica (0101020040).
Ações preventivas individuais	Aplicação de cariostático (por dente) (0101020058); Aplicação de selante (por dente) (0101020066); Aplicação tópica de flúor (individual por sessão) (0101020074); Evidenciação de placa bacteriana (0101020082).
Procedimentos restauradores	Restauração de dente decíduo (0307010023); Restauração de dente permanente anterior (0307010031); Restauração de dente permanente posterior (0307010040).
Procedimentos endodônticos	Obturação em dente permanente birradicular (0307020045); Obturação em dente permanente com três ou mais raízes (0307020053); Obturação em dente permanente unirradicular (0307020061); Obturação de dente decíduo (0307020037); Retratamento endodôntico em dente permanente birradicular (0307020088); Retratamento endodôntico em dente permanente com três ou mais raízes (0307020096); Retratamento endodôntico em dente permanente unirradicular (0307020053).
Procedimentos exodônticos	Exodontia de dente decíduo (0414020120); Exodontia de dente permanente (0414020138); Exodontia múltipla com alveoloplastia por sextante (0414020146).
Procedimentos periodontais	Raspagem, alisamento e polimento supragengivais (por sextante) (0307030016); Raspagem e alisamento subgengivais (por sextante) (0307030024); Raspagem coronorradicular (por sextante) (0307030032); Raspagem, alisamento e polimento supragengivais (por sextante) (0307030059).
Procedimentos protéticos	Prótese parcial mandibular removível (0701070099); Prótese parcial maxilar removível (0701070102); Prótese temporária (0701070110); Prótese total mandibular (0701070129); Prótese total maxilar (0701070137); Próteses coronárias/intrarradiculares fixas/adesivas (por elemento) (0701070145); Prótese dentária sobre implante (0701070153).

Fonte: Sistema de Informações Ambulatoriais do Sistema Único de Saúde (SIA/SUS).

Foram utilizados dados do SIA/SUS, disponibilizados na página eletrônica do Departamento de Informática do SUS (Datasus),^15^ considerando-se as produções anuais de janeiro a dezembro. O SIA/SUS dispõe de todos os procedimentos de saúde realizados nas unidades de saúde do Brasil. Foram extraídos dados sobre o tamanho populacional (Censo Demográfico de 2010) e estimativas intercensitárias, estas disponibilizadas pelo Instituto Brasileiro de Geografia e Estatística (IBGE), na Tabela 6579 do Sistema IBGE de Recuperação Automática,^16^ referente à população residente estimada, que utiliza o dia 1º de julho como referência anual.

O desfecho do estudo consistiu das taxas anuais de procedimentos por 100 mil habitantes, calculadas pelo quociente entre o número de procedimentos apresentados por região - em cada categoria, por ano - e a população residente estimada (Censo 2010 e projeções intercensos) da região para o mesmo ano, multiplicado pela constante de 100 mil.

A tabulação dos dados, o cálculo de taxas e proporções e a confecção dos gráficos e mapas foram realizados utilizando-se o programa Microsoft Excel. A análise descritiva baseou-se no cálculo de taxas e proporções.

A análise da série temporal foi realizada pelo método de regressão linear generalizada de Prais-Winsten, por se tratar de um procedimento delineado para quando a autocorrelação serial, advinda da dependência temporal, influenciar os dados, o que é frequente em medidas de dados populacionais.^17^ Na análise de regressão, foram construídos modelos em que a variável dependente foi o logaritmo das taxas de procedimentos; e a variável independente, os anos da realização dos procedimentos. Obteve-se o coeficiente β_1_ de inclinação da reta, o intervalo de confiança de 95% (IC_95%_) e o p-valor (pelo teste t da regressão), para avaliação da significância estatística. Nesta análise, empregou-se o pacote estatístico Stata® versão 15.0. A variação percentual anual (VPA) do período total, para cada grupo de procedimentos e seus respectivos IC_95%_, foi calculada utilizando-se, respectivamente, as seguintes equações:^17^

$$VPA=(1+10^{b1})*100\%$$

$${IC}_{95\%}=(-1+10^{b1mín})*100\%;(-1+10^{b1máx})*100\%$$

A tendência da produção odontológica foi considerada crescente quando o coeficiente da regressão foi positivo e p<0,05; decrescente, quando o coeficiente da regressão foi negativo e p<0,05; ou estacionária, quando não houve significância estatística.

Em virtude de estes estudo utilizar dados secundários de domínio público, não individualizados, foi dispensado da aprovação de Comitê de Ética em Pesquisa e da proposição de Termo de Consentimento Livre e Esclarecido.

## RESULTADOS

No período de 2008 a 2018, foram registrados 2,64 bilhões de procedimentos odontológicos, sendo o ano de 2010 o de maior produção, e o de 2018, o de menor produção. Em 2008, as taxas globais de procedimentos foram de 135.385 por 100 mil hab. na região Norte, e de 159.555 por 100 mil hab. na região Nordeste. Em 2018, as taxas de realização de procedimento foram de 34.666 por 100 mil hab. na região Nordeste e de 70.441 por 100 mil hab. na região Sul.

**Figura 1:**
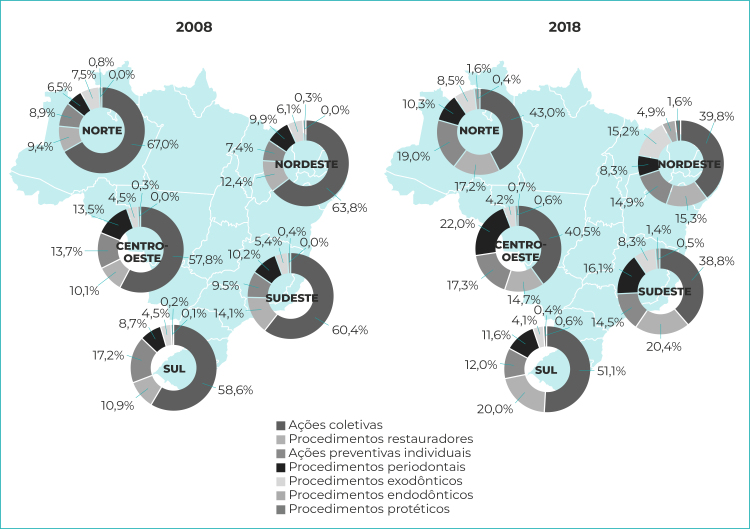
- Proporção das categorias de procedimentos odontológicos realizados, Brasil e regiões geopolíticas, 2008-2018

Segundo análise das categorias de procedimentos por região, as ações coletivas reduziram-se proporcionalmente, em todas as regiões brasileiras, entre os anos extremos da série histórica (Figura 1).

**Figura 2:**
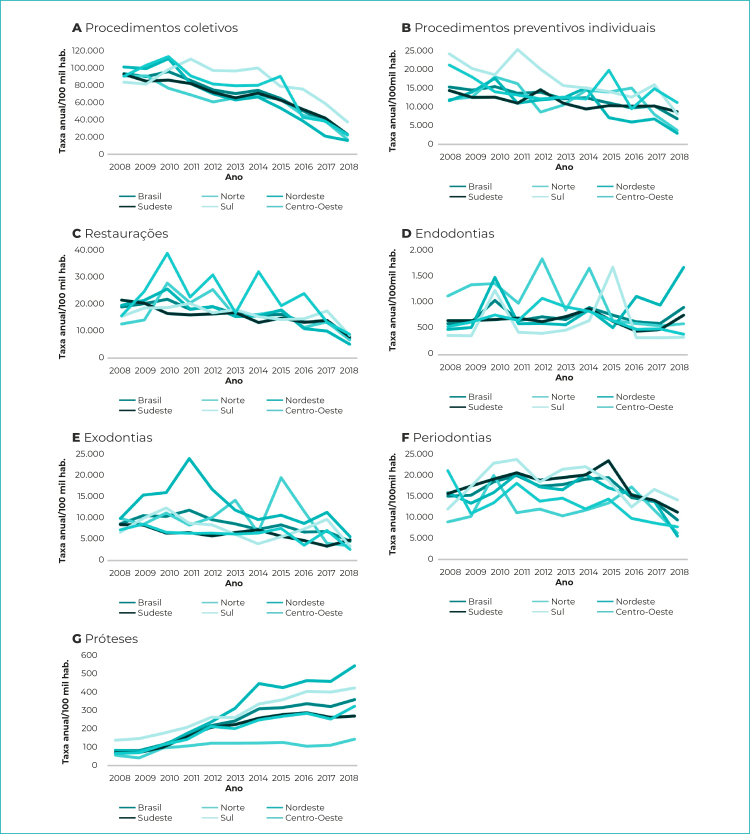
- Taxas médias anuais dos grupos de procedimentos odontológicos (por 100 mil habitantes), Brasil e regiões geopolíticas, 2008-2018

Observou-se tendência de estabilização nos procedimentos odontológicos até os anos de 2014 e 2015, com subsequente diminuição, não linear, na produção a partir de 2016, à exceção da categoria de procedimentos protéticos e endodônticos (Figura 2).

A análise de tendência revelou um decréscimo significativo da produção de ações coletivas no Brasil (VPA= -13,5; IC_95%_ -21,1;-5,2) e em suas regiões Norte (VPA= -14,4; IC_95%_ -21,1;-5,1), Nordeste (VPA= -17,8; IC_95%_ -25,6;-9,3), Sudeste (VPA= -12,7; IC_95%_ -19,8;-5,0) e Centro-Oeste (VPA= -12,8; IC_95%_ -20,6;-4,3) (Tabela 1).

**Tabela 1:** - Coeficiente de regressão e variação percentual anual das taxas médias dos procedimentos odontológicos (por 100 mil habitantes), Brasil e regiões geopolíticas, 2008-2018

Procedimentos e regiões	Taxa média (procedimentos por 100 mil hab.)	Coeficiente β_1_^a^ (IC_95%_^b^)	VPA^c^ % (IC_95%_^b^)	p-valor^d^	Tendência
Ações coletivas					
Norte	61.645,8	-0,07 (-0,10;-0,02)	-14,4 (-21,1;-5,1)	0,008	Redução
Nordeste	65.064,2	-0,09 (-0,13;-0,04)	-17,8 (-25,6;-9,3)	0,002	Redução
Sudeste	66.145,4	-0,06 (-0,10;-0,02)	-12,7 (-19,8;-5,0)	0,005	Redução
Sul	83.444,6	-0,03 (-0,08;0,01)	-7,6 (-16,0;1,6)	0,092	Estabilidade
Centro-Oeste	75.436,5	-0,06 (-0,10;-0,02)	-12,8 (-20,6;-4,3)	0,009	Redução
BRASIL	68.645,9	-0,06 (-0,10;-0,02)	-13,5 (-21,1;-5,2)	0,006	Redução
Ações preventivas individuais					
Norte	12.243,2	-0,04 (-0,09;-0,01)	-8,4 (-18,6;3,1)	0,127	Estabilidade
Nordeste	10.694,5	-0,06 (-0,09;-0,02)	-11,9 (-18,5;-4,8)	0,005	Redução
Sudeste	11.493,7	-0,02 (-0,02;-0,01)	-3,9 (-5,5;-2,3)	<0,001	Redução
Sul	17.524,5	-0,03 (-0,05;-0,02)	-7,4 (-9,9;-5,0)	<0,001	Redução
Centro-Oeste	14.615,4	-0,02 (-0,03;0,00)	-3,5 (-7,0;0,0)	0,052	Estabilidade
BRASIL	12.437,1	-0,03 (-0,03;-0,02)	-6,2 (-7,7;-4,8)	<0,001	Redução
Restaurações					
Norte	16.654,4	-0,03 (-0,06;0,01)	-6,1 (-13,7;2,2)	0,128	Estabilidade
Nordeste	16.430,0	-0,05 (-0,08;-0,02)	-11,0 (-16,5;-5,1)	0,003	Redução
Sudeste	15.680,5	-0,03 (-0,03;-0,02)	-5,8 (-7,4;-4,1)	<0,001	Redução
Sul	16.338,8	-0,02 (-0,03;-0,01)	-3,9 (-6,3;-1,4)	0,006	Redução
Centro-Oeste	22.735,9	-0,03 (-0,06;0,01)	-6,3 (-13,4;1,4)	0,095	Estabilidade
BRASIL	16.582,5	-0,03 (-0,05;-0,02)	-7,3 (-10,5;-3,9)	0,001	Redução
Endodontias					
Norte	1.050,4	-0,04 (-0,07;-0,02)	-9,6 (-14,6;-4,2)	0,003	Redução
Nordeste	844,1	0,03 (0,00;0,06)	7,1 (-0,6;15,4)	0,066	Estabilidade
Sudeste	644,0	0,00 (-0,03;0,02)	-1,1 (-6,1;4,2)	0,650	Estabilidade
Sul	580,9	-0,01 (-0,06;0,05)	-1,9 (-13,8;11,8)	0,752	Estabilidade
Centro-Oeste	660,6	-0,02 (-0,06;0,03)	-3,7 (-12,6;6,2)	0,410	Estabilidade
BRASIL	724,7	0,00 (-0,01;0,02)	0,4 (-3,2;4,1)	0,803	Estabilidade
Exodontias					
Norte	9.637,6	-0,03 (-0,09;0,02)	-7,3 (-18,5;5,5)	0,218	Estabilidade
Nordeste	12.713,0	-0,03 (-0,07;0,00)	-7,4 (-14,4;0,3)	0,056	Estabilidade
Sudeste	5.890,6	-0,03 (-0,05;-0,02)	-7,1 (-10,8;-3,4)	0,002	Redução
Sul	7.240,5	-0,03 (-0,07;0,01)	-6,6 (-14,3;1,9)	0,110	Estabilidade
Centro-Oeste	5.966,1	-0,03 (-0,04;-0,02)	-6,0 (-8,3;-3,6)	<0,001	Redução
BRASIL	8.303,2	-0,03 (-0,05;-0,01)	-6,9 (-10,5;-3,1)	0,003	Redução
Periodontias					
Norte	12.007,9	-0,01 (-0,04;0,03)	-1,2 (-8,1;6,1)	0,705	Estabilidade
Nordeste	15.575,7	-0,03 (-0,08;0,02)	-7,6 (-17,7;3,9)	0,161	Estabilidade
Sudeste	17.814,2	-0,01 (-0,04;0,02)	-3,0 (-9,2;3,6)	0,322	Estabilidade
Sul	18.254,1	0,00 (-0,03;0,03)	-0,7 (-7,1;6,1)	0,805	Estabilidade
Centro-Oeste	13.089,8	-0,03 (-0,05;-0,01)	-6,7 (-10,8;-2,4)	0,007	Redução
BRASIL	16.418,6	-0,02 (-0,05;0,01)	-4,2 (-11,0;3,2)	0,228	Estabilidade
Próteses					
Norte	102,1	0,04 (0,01;0,07)	9,5 (1,5;18,2)	0,024	Aumento
Nordeste	304,5	0,09 (0,05;0,12)	22,3 (12,8;32,5)	<0,001	Aumento
Sudeste	198,0	0,06 (0,02;0,10)	15,3 (5,6;25,9)	0,005	Aumento
Sul	284,6	0,05 (0,04;0,06)	12,8 (9,8;16,0)	<0,001	Aumento
Centro-Oeste	198,0	0,07 (0,04;0,10)	17,8 (9,8;26,5)	0,001	Aumento
BRASIL	231,6	0,07 (0,04;0,10)	16,9 (9,1;25,2)	0,001	Aumento

a) Após transformação logarítmica das taxas; b) IC95%: intervalo de confiança de 95%; c) VPA: variação percentual anual; d) Teste t da regressão linear de Prais-Winsten, nível de significância de 5%.

A região Norte apresentou a menor taxa média do período para essa categoria (61,6 mil/100 mil hab.). Por sua vez, a região Sul apresentou a maior taxa média de procedimentos (83,4 mil/100 mil hab.) e tendência estacionária (p=0,092). O Brasil registrou uma média de 68,6 mil procedimentos coletivos por 100 mil hab. para o período (Tabela 1).

As ações preventivas individuais apresentaram tendência decrescente no Brasil (VPA= -6,2; IC_95%_ -7,7;-4,8), nas regiões Nordeste (VPA= -11,9; IC_95%_ -18,5;-4,8), Sudeste (VPA= -3,9; IC_95%_ -5,5;-2,3) e Sul (VPA= -7,4; IC_95%_ -9,9;-5,0); e estabilidade nas demais regiões. Ademais, a maior e a menor taxa média de procedimentos preventivos individuais por 100 mil hab. nos 11 anos selecionados foram registradas nas regiões Sul (17,5 mil) e Nordeste (10,7 mil) respectivamente. A média global de procedimentos preventivos individuais no Brasil foi de 12,4 mil por 100 mil hab. (Tabela 1).

Observaram-se oscilações nas taxas médias dos procedimentos restauradores nas regiões Norte e Centro-Oeste, entre 2008 e 2018 (Figura 2). O Brasil (VPA= -7,3; IC_95%_ -10,5;-3,9) e as regiões Nordeste (VPA= -11,0; IC_95%_ -16,5;-5,1), Sudeste (VPA= -5,8; IC_95%_ -7,4;-4,1) e Sul (VPA= -3,9; IC_95%_ -6,3;-1,4) apresentaram tendência temporal declinante.

A taxa média de restaurações foi de 16,6 mil por 100 mil hab. no Brasil, observando-se nível mais elevado na região Centro-Oeste (22,7 mil por 100 mil hab.) e menor na região Sudeste (15,7 mil por 100 mil hab.).

A categoria de procedimentos endodônticos foi a que obteve a segunda menor taxa média de realização de procedimentos na série histórica. No ano de 2010, as regiões Nordeste e Norte apresentaram as maiores taxas (Figura 2) desse grupo de procedimentos. A região Norte apresentou tendência decrescente na realização destes procedimentos (VPA= -9,6; IC_95%_ -14,6;-4,2), enquanto, nas demais regiões e no país, a tendência foi estacionária (Tabela 1).

As taxas médias de procedimentos de exodontias apresentaram tendência declinante para o Brasil (VPA= -6,9; IC_95%_ -10,5;-3,1), nas regiões Sudeste (VPA= -7,1; IC_95%_ -10,8;-3,4) e Centro-Oeste (VPA= -6,0; IC_95%_ -8,3;-3,6), enquanto mantiveram-se estacionárias nas demais regiões (Tabela 1). As maiores oscilações nas taxas médias desse grupo de procedimentos foram observadas nas regiões Norte e Nordeste, tendo esta última permanecido com médias superiores à média nacional, ao longo de toda a série histórica (Figura 2), e obtido a maior taxa média (12,7 mil procedimentos por 100 mil hab.) para os 11 anos do estudo.

No grupo de procedimentos periodontais, a região Centro-Oeste foi a única a apresentar tendência declinante (VPA= -6,7; IC_95%_ -10,8;-2,4); nas demais regiões e no Brasil como um todo, as taxas mantiveram-se estáveis (p>0,05) (Tabela 1).

A categoria de procedimentos protéticos apresentou as menores taxas médias. De 2008 a 2018, o Brasil realizou, em média, 231,6 procedimentos protéticos por 100 mil habitantes. Esse foi o único grupo de procedimentos a apresentar tendência ascendente em todas as regiões e no Brasil (Tabela 1). A maior variação percentual anual foi observada na região Nordeste, com aumento de 22,3% (IC_95%_ 12,8;32,5).

## DISCUSSÃO

A análise mostrou tendência de diminuição global, bem como de diversos grupos de procedimentos odontológicos no Brasil, no âmbito do SUS, no período de 2008 a 2018. À exceção das próteses, que apresentaram tendência crescente no país e em todas as suas regiões geopolíticas, observou-se tendência decrescente significativa na realização dos grupos de procedimentos relativos às ações coletivas, ações preventivas individuais, restaurações e exodontias. Este declínio mostra-se ainda maior nas regiões Norte e Nordeste.

Em 2008, a soma das ações coletivas e preventivas individuais aproximava-se de 70% em cada região. Em 2018, além de redução quantitativa dessas ações, houve diminuição proporcional desses dois grupos de procedimentos, indicando que os cirurgiões-dentistas passaram a dedicar mais tempo aos procedimentos clínicos curativos, frente aos coletivos-preventivos, na comparação com 2008. Mudanças em ambos os grupos de procedimentos no Brasil, embora de tendências contrárias, foram observadas em dois estudos anteriores, segundo os quais registrou-se aumento nessa produção entre 1994 e 2007,^13^ e declínio entre 1999 e 2017.^14^

A região Nordeste, que concentrava as maiores taxas de procedimentos anuais em 2008, sofreu redução desses serviços em 2018, enquanto a região Sul teve a maior produção entre as regiões no ano de 2018, porém com produção muito baixa comparativamente aos anos iniciais da série. Neves et al.^18^ observaram importantes diferenças entre as regiões na realização de procedimentos odontológicos curativos, tendo o Sul e o Sudeste apresentado as maiores prevalências, enquanto o Norte e o Nordeste as menores. No estudo mencionado, a maior prevalência de procedimentos curativos mostrou-se associada à presença de materiais e instrumentais odontológicos, acolhimento, continuidade do cuidado, visita domiciliar realizada pelo cirurgião-dentista e equipe de Saúde Bucal da modalidade II. Apesar de as equipes serem presentes e atuantes, faltaram-lhes condições físicas ou materiais para executar alguns procedimentos.

As maiores taxas de procedimentos coletivos e preventivos individuais foram registradas em 2010, considerando-se o Brasil como unidade. Ao se avaliar a evolução da escovação dental supervisionada (procedimento coletivo) na série histórica de 2008 a 2017, Chaves et al.^19^ apontaram que 2010 foi o ano com o maior número dessas ações no país, fato relacionado à distribuição, em 2009, de 40,6 milhões de *kits* de saúde bucal pelo Ministério da Saúde em 4.597 municípios.

A região Nordeste destacou-se por apresentar, no ano inicial da série, a maior taxa de ações coletivas e preventivas individuais, e no ano final, a menor taxa para os mesmos procedimentos. Além disso, a soma de procedimentos preventivos coletivos e individuais caiu para menos de 50% do total da produção nessa região. Corrobora esse achado o estudo de Silva et al.^20^ ao observarem que, no biênio 2015-2017, a produção ambulatorial de procedimentos curativos superou a de procedimentos preventivos no Nordeste.

No presente estudo, entre 2008 e 2018, identificou-se tendência de diminuição significativa das taxas de procedimentos periodontais no Centro-Oeste, e tendência estacionária nas demais regiões. No estudo sobre os anos de 1999 a 2017, os procedimentos periodontais apresentaram tendência linear de crescimento em todas as regiões brasileiras,^14^ possivelmente acentuada pelo aumento do registro desse grupo de procedimentos entre 2008 e 2012, ao passo que, nos anos iniciais da série (1999 a 2007), eram pouco registrados.

Outros resultados do presente trabalho mostraram-se diferentes dos achados de Chisini et al.,^14^ dada a diferença entre os intervalos de anos analisados. O fato de os autores do estudo citado considerarem o período antes da inserção das equipes de Saúde Bucal na ESF, sua inserção a partir de 2002 e o aumento expressivo dessas equipes e de CEOs, provocado pela PNSB a partir de 2004, culminou em desfecho ascendente para alguns procedimentos odontológicos, não verificado aqui.

Voltando a este estudo e sua série histórica, as maiores taxas médias de exodontias e endodontias referiram-se às regiões Nordeste e Norte, e as maiores taxas de restaurações, à região Centro-Oeste. Os resultados do levantamento epidemiológico SB Brasil 2010 apontaram o Centro-Oeste, o Norte e o Nordeste com mais indivíduos necessitados de tratamentos exodônticos, endodônticos e restauradores,^8^^)^ coincidindo com os procedimentos que estão sendo ofertados pelo SUS.

O decréscimo na produção em saúde bucal no Brasil foi acompanhado pela redução dos investimentos federais na atenção básica, no início de 2013, sua estabilização entre 2013 e 2016, e nova redução em 2017.^19^^),(^^21^ Soma-se a isso a promulgação da Emenda Constitucional (EC) n^o^ 55, de 15 de dezembro de 2016, responsável pelo congelamento dos gastos sociais por 20 anos, legitimando novas medidas de austeridade fiscal e a abertura de um caminho institucionalizado para a redução dos investimentos na saúde pública, e, como consequência, tendo impacto negativo nas políticas de saúde bucal.^21^^),(^^22^

Outro fator relacionado ao período analisado são as sucessivas mudanças na gestão do Ministério da Saúde e na Coordenação-Geral de Saúde Bucal que, desde 2015, expressam a instabilidade política no país e geram efeitos na implementação do Programa Brasil Sorridente (PNSB).^19^^),(^^22^

O e-SUS AB, um sistema de informações implantado pelo Departamento de Atenção Básica do Ministério da Saúde a partir de 2013, poderia alavancar o registro de procedimentos no país. Entretanto, o novo sistema não se refletiu em variações positivas nas taxas de consultas e procedimentos notificados no SIA/SUS.^23^ Se, todavia, são poucos os estudos sobre a interferência do e-SUS no registro de dados, pesquisas apontam dificuldades na utilização e alimentação das informações de saúde por esse sistema no cotidiano de trabalho dos profissionais,^24^^),(^^25^ o que contribui para o registro incorreto e subnotificações.^26^ Sendo assim, deve-se refletir sobre o potencial de contribuição do e-SUS para o aprimoramento do registro das ações de saúde.

Se a inclusão do sistema e-SUS é colocada como uma das prováveis causas da redução dos indicadores de produtividade de saúde bucal no país, também o são as reduções no repasse federal e dificuldades de cofinanciamento pelo nível local, em função de diminuições na arrecadação e sobrecarga municipal no financiamento da Saúde Pública.^19^ Chaves et al. reafirmam que as causas para essa variação precisam ser investigadas por estudos de análise da implantação e resultados da política de saúde bucal nos âmbitos regional e municipal. 

O aumento observado na proporção de procedimentos protéticos, em todas as regiões do país, deve ser visto com cautela, pois ainda representa percentuais muito baixos perante as demais categorias de procedimentos. As maiores taxas de procedimentos protéticos foram registradas no Nordeste, situação encontrada também por Chisini et al.^14^ entre 1999 e 2017, resultado tanto de investimento nos laboratórios regionais de prótese dentária como do fato de a região apresentar maior população SUS-dependente.^14^ O estudo de Aguiar e Celeste^27^ evidenciou contradição entre a maior prevalência de edêntulos concentrar-se na região Sudeste e a maior quantidade de laboratórios regionais de próteses por habitante localizar-se na região Nordeste. As regiões com maior acesso a esses serviços (salvo para prótese fixa) foram aquelas com maior disponibilidade de tais laboratórios.^27^

A alta demanda acumulada por procedimentos protéticos e o envelhecimento populacional são fatores a serem considerados, visando ao aumento do investimento em reabilitação protética. Dados do SB Brasil 2010 mostraram que 92,7% dos idosos e 68,8% dos adultos necessitavam de algum tipo de prótese dentária.^8^ A inclusão da reabilitação protética na atenção básica (nas unidades de Saúde da Família) e secundária (nos CEOs), a implantação dos laboratórios regionais de prótese dentária desde 2005 e a contratação de laboratórios privados são fatores primordiais para a tendência ascendente desse grupo de procedimentos.^4^ Entre 2011 e 2014, houve aumento na identificação, pelas equipes de Saúde Bucal, de pessoas com necessidades de próteses dentárias, e consequente aumento nos procedimentos protéticos; contudo, a frequência de fornecimento de próteses dentárias ainda é considerada muito baixa no Brasil.^28^ No período de 2003 a 2014, os repasses financeiros à atenção especializada em saúde apresentaram aumento; porém, de 2015 a 2017, houve manutenção nos valores destinados a esse nível de assistência.^21^

O crescimento no total de equipes de Saúde Bucal no Brasil, entre 2003 e 2017,^19^ é uma informação conflitante com a redução nas taxas de procedimentos observada no presente estudo, entre 2008 e 2018, sugerindo possíveis limitações no planejamento, implantação e acompanhamento gerencial do trabalho dessas equipes. Porém, o número crescente das equipes de ESF, com uma pequena redução apenas em 2016, não se refletiu na ampliação da cobertura populacional^19^ e, portanto, merece estudos futuros. 

As limitações deste estudo, inerentes a pesquisas com dados secundários, remetem à qualidade e cobertura dos registros dos sistemas de informações, passíveis de erros e subnotificações. Como pontos positivos, destacam-se seu caráter abrangente, a utilização de dados do Ministério da Saúde e a possibilidade de replicação da metodologia utilizada em outras áreas geográficas e períodos. Ademais, a análise das categorias dos procedimentos odontológicos repercute na organização, oferta e qualificação da assistência odontológica.

Conclui-se que a produção ambulatorial odontológica no SUS apresentou decréscimo significativo no Brasil entre 2008 e 2018, acentuadamente após 2015, com variações entre regiões e grupos de procedimentos. Os únicos procedimentos com tendência ascendente, no período analisado, foram os protéticos, no Brasil como um todo, ou em suas regiões geopolíticas separadamente. Os resultados deste estudo ajudam a entender a evolução da produção ambulatorial no país e, assim, podem auxiliar na gestão de políticas que incluem a saúde bucal. Percebe-se a necessidade de aprimorar o planejamento e o monitoramento permanente das ações, além de maiores investimentos na área da saúde bucal, para que mais brasileiros possam ter acesso às ações e serviços de saúde.
