# Supplementary material for: Time series of the production of dental procedures in the Brazilian National Health System, Brazil, 2008-2018
Source: Epidemiol Serv Saude. 2022 Mar 21;31(1):e2021213. [Article in Portuguese] doi: 10.1590/S1679-49742022000100007 (PMC11524155; doi:10.1590/S1679-49742022000100007)
Supplement: Translation

# Translation: Time series of the production of dental procedures in the Brazilian National Health System, Brazil, 2008-2018

**Authors:** Georgia Costa de Araújo Souza, Sandro Alves Mourão, Gustavo Barbalho Guedes Emiliano

**Affiliations:** 1 Universidade Federal do Rio Grande do Norte, Departamento de Odontologia, Natal, RN, Brazil; 2 Universidade do Estado do Rio Grande do Norte, Departamento de Odontologia, Caicó, RN, Brazil

**Keywords:** Oral Health, Information Systems, Public Health Surveillance, Outpatient Care, Time Series Studies.

## Abstract

**Objective:**

To analyze dental procedures provided by the Brazilian National Health System (SUS) in Brazil and its macro-regions, between 2008 and 2018.

**Methods:**

This was a time series study using data from SUS Outpatient Information System. Annual and overall rates of dental procedures (per 100,000 inhabitants), according to the categories of dental procedures and regions were calculated. Prais-Winsten regression was used to analyze time trends, while annual percentage change (APC) was calculated.

**Results:**

Decreasing trends were found in Brazil, in collective measures (APC= -13.5%; 95%CI -21.1;-5.2), individual preventive measures (APC= -6.2%; 95%CI -7.7;-4.8), dental restoration (APC= -7.3%; 95%CI -10.5;-3.9) and tooth extraction procedures (APC= -6.9; 95%CI -10,5;-3,1). Endodontics and periodontics showed stationary trend in most regions and Brazil. Prosthetic procedures showed an upward trend in all regions and Brazil (APC= 16.9%; 95%CI 9.1;25.2).

**Conclusion:**

Dental procedures in the SUS decreased between 2008-2018; with the exception of prosthetic procedures, which showed a rising trend.

## INTRODUCTION

**Table 1:** - Regression coefficient and annual percentage change of the average rates of dental procedures (per 100,000 inhabitants), Brazil and its macro-regions, 2008-2018

Study contributions	
Main results	Dental procedures in the SUS decreased between 2008-2018; with the exception of prosthetic procedures, which showed an upward trend.
Implications for services	The study can help dental professionals and managers identify the decrease in dental procedures provided, stimulating the monitoring of dental production at local level.
Perspectives	Search for macro and micro solutions to meet the demands for dental services, given the downward trend in their production. Future studies should analyze dental production, investment, human resources and demand for dental services in the SUS.

The inclusion of Oral Health teams in the Family Health Strategy (FHS) provided the reorganization of oral health services in primary health care and greater provision of dental procedures via the Brazilian National Health System (SUS).^1^ It could be seen that access to oral health has increased among the most vulnerable population, despite the difficulties faced by the system, such as the fragmentation of health policies and programs, management and social control qualification, in addition to the difficulty in the organization of a regionalized and hierarchical network of health actions and services, compromising the universality and integrality of the SUS.^2^ Since the establishment of the National Oral Health Policy (PNSB) guidelines, in 2004, important advances in oral health care have been observed, while new challenges have arisen.^3^

Among the advances achieved, we can highlight the greatest incentive for the implementation of programmatic actions aimed at fluoridation of public water supply, the structuring of a reference network and counter-reference in Dental Specialty Centers (DSC) and the Regional Dental Prosthetic Laboratories (RDPL), and a 390% increase in the number of Oral Health teams in the FHS.^4^

Moreover, the expansion of secondary health care, promoted by the PNSB, have incorporated the offer of more complex procedures, such as prosthetic, endodontic and orthodontic treatments, diagnostic support (radiology and biopsy) among others, reorganizing oral health care network.^4^

The adoption of the measures proposed by the PNSB, however, has not occurred uniformly throughout the country.^5^ There is, therefore, a need to think about the application of PNSB guidelines locoregionally, associated to other public policies, in order to promote the implementation of intersectoral actions, greater access to health actions and services in an integral way and overcoming social inequalities.^6^ Epidemiology is responsible for providing information on oral health profile of the population, service organization and health care planning.^7^

Data from the last national oral health survey, SB Brazil 2010, showed that the majority of the Brazilian population aged 12 (58.1%) and 15 to 19 years (46.3%) sought the public health system for the use of dental services. Treatment (33.1% and 37.3%, respectively), routine dental check-ups or prevention (38.3% and 36.2%, respectively) were the most frequently mentioned reasons for the last dental consultation. As for adults and the elderly, the majority used the private service for the last dental consultation (49.1% and 59.8%, respectively), and the most common reasons were (i) the need for treatment (44.6%) and prevention (21.4%) among adults, and (ii) treatment (36.8%) and tooth extraction (26.9%) among the elderly.^8^ These data reaffirm oral health care characteristics, person-centered care and technical clinical practices, even in the FHS, justified by the lack of robust, comprehensive and coordinated strategies for coping with substantial pent-up demand.^9^ It can be taken into consideration that knowledge about the profile of dental procedures provided can contribute to the analysis of dental care characteristics. Moreover, the understanding of the temporal trend of dental procedures has allowed the identification of changes in oral health care over the years.^10^

There are evaluations of the evolution of dental care at local and regional levels^10^^)-(^^12^, but there are few studies at national level^13^^).(^^14^ that have investigated since the period prior to the inclusion of oral health teams in the FHS, including investments related to the PNSB in 2004, and showed an upward trend in dental procedures in Brazil. Depending on the time interval and context analyzed, the results of these studies may differ according to the population profile and guidelines for public policies. The aim of this study was to analyze the performance of dental procedures in the Brazilian National Health System, in Brazil and its macro-regions, between 2008 and 2018.

## METHODS

This was an ecological time series study. The units of analysis were Brazil and its five macro-regions: North, Northeast, Southeast, South and Midwest. The period investigated comprised the years 2008 to 2018. The beginning of this period coincided with the change in the SUS Outpatient Information System (SIA/SUS), when the nomenclature for dental procedures and their respective codes was updated in the table of SUS procedures.^15^ In this study, the table of SUS outpatient dental procedures were included.

The categories of dental procedures were the study variables. Their definitions and other information are found in Box 1: collective measures; individual preventive measures; dental restorations; endodontics; tooth extraction; periodontics; prosthetic procedures; in addition to the population, years and the geography of Brazil and its macro-regions.

**Box 1:** - Categories of dental procedures analyzed and their respective codes in the SUS Outpatient Information System (SIA/SUS)

Collective measures	Educational activity/guidelines for groups in primary care (0101010010); Collective measures of topical application of fluoride gel (0101020015); Collective measures of fluoride mouthwash (0101020023); Collective measures of supervised toothbrushing (0101020031); Collective measures of oral examination with epidemiological purpose (0101020040).
Individual preventive measures	Application of cariostatic (per tooth) (0101020058); Dental sealant application (per tooth) (0101020066); Topical application of fluoride (individual per session) (0101020074); Evidence of dental plaque (0101020082).
Dental restoration procedures	Restoration of deciduous tooth (0307010023); Restoration of anterior permanent tooth (0307010031); Restoration of posterior permanent tooth (0307010040).
Endodontic procedures	Biradicular permanent tooth filling (0307020045); Permanent tooth with three or more roots filling (0307020053); One-rooted permanent tooth filling (0307020061); Deciduous tooth filling (0307020037); Endodontic retreatment of two-rooted permanent tooth (0307020088); Endodontic retreatment of permanent tooth with three or more roots (0307020096); Endodontic retreatment in one-rooted permanent tooth (0307020053).
Tooth extraction procedures	Deciduous tooth extraction (0414020120); Permanent tooth extraction (0414020138); Multiple tooth extraction with alveoloplasty per sextant (0414020146).
Periodontic procedures	Supragingival scraping, straightening and polishing (per sextant) (0307030016); Subgingival scraping and straightening (by sextant) (0307030024); Coronoradicular scraping (per sextant) (0307030032); Scraping, straightening and supragingival polishing (by sextant) (0307030059).
Prosthetic procedures	Mandibular removable partial denture (0701070099); Maxillary removable partial denture (0701070102); Temporary prosthesis (0701070110); Total mandibular denture (0701070129); Total maxillary denture (0701070137); Fixed/adhesive coronary/intraradicular dental prostheses (by element) (0701070145); Dental implant prosthetic (0701070153).

Source: SUS Outpatient Information System (SIA/SUS).

Data from SIA/SUS, available on the IT Department of the Brazilian National Health System (DATASUS) website, were used,^15^ taking into consideration the annual productions from January to December. On the SIA/SUS we can find all the health procedures performed in health centers in Brazil. Data on population size (2010 Census) and intercensal estimates were obtained. They were made available by the Instituto Brasileiro de Geografia e Estatística (IBGE), in Table 6579 of the IBGE System of Automatic Recovery,^16^ for the estimated resident population, which uses July 1 as the annual reference date.

The outcome of this study consisted of annual rates of procedures per 100,000 inhabitants, calculated by the quotient between the number of procedures presented by region - in each category, per year - and the estimated resident population (2010 Census and inter-census projections) of the region for the same year, multiplied by the constant of 100,000.

Tabulation of the data, calculation of rates and proportions and preparation of the graphs and maps were performed using the Microsoft Excel. Descriptive analysis was based on the calculation of rates and proportions.

Temporal series analysis was performed using the Prais-Winsten generalized linear regression method, as it is a procedure designed for the serial autocorrelation, resulting from temporal dependence, whenever it influences the data, which occurs frequently during population data measurement.^17^ Regarding the regression analysis, models were constructed in which the dependent variable was the logarithm of procedure rates; and the independent variable, the years when the procedures were performed. The coefficient β1 of the slope of the regression line, 95% confidence interval (95%CI) and p-value (using the linear regression t-test) were obtained to evaluate the statistical significance. The Stata® statistical software package, version 15.0 was used in this analysis. The annual percentage change (APC) of the total period for each group of procedures and their respective 95%CI was calculated using the following equations, respectively:^17^

$$APC\;=\;(1\;+\;10^{b1})\;*\;100\%$$

$$95\%CI\;=\;(-1+10^{b1mín})*100\%;(-1+10^{b1máx})*100\%$$

Dental production was considered having an upward trend when the regression coefficient was positive and the p-value <0.05; a decreasing trend when the regression coefficient was negative and p-value <0.05; and a stationary trend when there was no statistical significance.

This study used secondary data in the public domain, not individualized, therefore it was exempted from the approval of a Research Ethics Committee and the proposition of a Free and Informed Consent Form.

## RESULTS

Between 2008 and 2018, 2.64 billion dental procedures were recorded. 2010 was the year with the highest production and 2018 registered the lowest production. In 2008, overall procedure rates were 135,385 per 100,000 inhabitants in the North region, and 159,555 per 100,000 inhabitants in the Northeast region. In 2018, procedure rates were 34,666 per 100,000 inhabitants in the Northeast region and 70,441 per 100,000 inhabitants in the South region.

According to the analysis of the categories of dental procedures by region, collective measures were proportionally reduced, in all Brazilian regions, between the extreme years of the historical series (Figure 1).

**Figure 1:** - Proportion of the categories of dental procedures performed, Brazil and its macro-regions, 2008-2018

There was a stabilization trend in dental procedures between 2014 and 2015, with subsequent nonlinear reduction in production as of 2016, except for the category of prosthetic and endodontic procedures (Figure 2).

**Figure 2:** - Annual average rates of dental procedure groups (per 100,000 inhabitants), Brazil and its macro-regions, 2008-2018

The trend analysis showed a significant decrease in the production of collective measures in Brazil (VPA= -13.5; 95%CI -21.1; -5.2) and its regions, North (VPA= -14.4; 95%CI -21.1;-5.1), Northeast (VPA= -17.8; 95%CI -25.6; -9.3), Southeast (VPA= -12.7; 95%CI -19.8;-5.0) and Midwest (VPA= -12.8; 95%CI -20.6;-4.3) (Table 1). The North region showed the lowest average rate in the period for this category (61.6 thousand/100,000 inhabitants), while the Southern region presented the highest average rate of dental procedures (83.4 thousand/100,000 inhabitants) and stationary trend (p=0.092). Brazil recorded an average of 68,600 collective procedures per 100,000 inhabitants for the period (Table 1).

**Table 1:** - Regression coefficient and annual percentage change of the average rates of dental procedures (per 100,000 inhabitants), Brazil and its macro-regions, 2008-2018

Procedures and regions	Average rates (procedures per 100,000 inhabitants)	Coefficient β_1_^a^ (95%CI^b^)	APC^c^ % (95%CI^b^)	p-value^d^	Trend
Collective measures					
North	61,645.8	-0.07 (-0.10;-0.02)	-14.4 (-21.1;-5.1)	0.008	Reduction
Northeast	65,064.2	-0.09 (-0.13;-0.04)	-17.8 (-25.6;-9.3)	0.002	Reduction
Southeast	66,145.4	-0.06 (-0.10;-0.02)	-12.7 (-19.8;-5.0)	0.005	Reduction
South	83,444.6	-0.03 (-0.08;0.01)	-7.6 (-16.0;1.6)	0.092	Stability
Midwest	75,436.5	-0.06 (-0.10;-0.02)	-12.8 (-20.6;-4.3)	0.009	Reduction
BRAZIL	68,645.9	-0.06 (-0.10;-0.02)	-13.5 (-21.1;-5.2)	0.006	Reduction
Individual preventive measures					
North	12,243.2	-0.04 (-0.09;-0.01)	-8.4 (-18.6;3.1)	0.127	Stability
Northeast	10,694.5	-0.06 (-0.09;-0.02)	-11.9 (-18.5;-4.8)	0.005	Reduction
Southeast	11,493.7	-0.02 (-0.02;-0.01)	-3.9 (-5.5;-2.3)	<0.001	Reduction
South	17,524.5	-0.03 (-0.05;-0.02)	-7.4 (-9.9;-5.0)	<0.001	Reduction
Midwest	14,615.4	-0.02 (-0.03;0.00)	-3.5 (-7.0;0.0)	0.052	Stability
BRAZIL	12,437.1	-0.03 (-0.03;-0.02)	-6.2 (-7.7;-4.8)	<0.001	Reduction
Dental restoration procedures					
North	16,654.4	-0.03 (-0.06;0.01)	-6.1 (-13.7;2.2)	0.128	Stability
Northeast	16,430.0	-0.05 (-0.08;-0.02)	-11.0 (-16.5;-5.1)	0.003	Reduction
Southeast	15,680.5	-0.03 (-0.03;-0.02)	-5.8 (-7.4;-4.1)	<0.001	Reduction
South	16,338.8	-0.02 (-0.03;-0.01)	-3.9 (-6.3;-1.4)	0.006	Reduction
Midwest	22,735.9	-0.03 (-0.06;0.01)	-6.3 (-13.4;1.4)	0.095	Stability
BRAZIL	16,582.5	-0.03 (-0.05;-0.02)	-7.3 (-10.5;-3.9)	0.001	Reduction
Endodontics					
North	1,050.4	-0.04 (-0.07;-0.02)	-9.6 (-14.6;-4.2)	0.003	Reduction
Northeast	844.1	0.03 (0.00;0.06)	7.1 (-0.6;15.4)	0.066	Stability
Southeast	644.0	0.00 (-0.03;0.02)	-1.1 (-6.1;4.2)	0.650	Stability
South	580.9	-0.01 (-0.06;0.05)	-1.9 (-13.8;11.8)	0.752	Stability
Midwest	660.6	-0.02 (-0.06;0.03)	-3.7 (-12.6;6.2)	0.410	Stability
BRAZIL	724.7	0.00 (-0.01;0.02)	0.4 (-3.2;4.1)	0.803	Stability
Tooth extraction procedures					
North	9,637.6	-0.03 (-0.09;0.02)	-7.3 (-18.5;5.5)	0.218	Stability
Northeast	12,713.0	-0.03 (-0.07;0.00)	-7.4 (-14.4;0.3)	0.056	Stability
Southeast	5,890.6	-0.03 (-0.05;-0.02)	-7.1 (-10.8;-3.4)	0.002	Reduction
South	7,240.5	-0.03 (-0.07;0.01)	-6.6 (-14.3;1.9)	0.110	Stability
Midwest	5,966.1	-0.03 (-0.04;-0.02)	-6.0 (-8.3;-3.6)	<0.001	Reduction
BRAZIL	8,303.2	-0.03 (-0.05;-0.01)	-6.9 (-10.5;-3.1)	0.003	Reduction
Periodontics					
North	12,007.9	-0.01 (-0.04;0.03)	-1.2 (-8.1;6.1)	0.705	Stability
Northeast	15,575.7	-0.03 (-0.08;0.02)	-7.6 (-17.7;3.9)	0.161	Stability
Southeast	17,814.2	-0.01 (-0.04;0.02)	-3.0 (-9.2;3.6)	0.322	Stability
South	18,254.1	0.00 (-0.03;0.03)	-0.7 (-7.1;6.1)	0.805	Stability
Midwest	13,089.8	-0.03 (-0.05;-0.01)	-6.7 (-10.8;-2.4)	0.007	Reduction
BRAZIL	16,418.6	-0.02 (-0.05;0.01)	-4.2 (-11.0;3.2)	0.228	Stability
Prosthesis					
North	102.1	0.04 (0.01;0.07)	9.5 (1.5;18.2)	0.024	Increase
Northeast	304.5	0.09 (0.05;0.12)	22.3 (12.8;32.5)	<0.001	Increase
Southeast	198.0	0.06 (0.02;0.10)	15.3 (5.6;25.9)	0.005	Increase
South	284.6	0.05 (0.04;0.06)	12.8 (9.8;16.0)	<0.001	Increase
Midwest	198.0	0.07 (0.04;0.10)	17.8 (9.8;26.5)	0.001	Increase
BRAZIL	231.6	0.07 (0.04;0.10)	16.9 (9.1;25.2)	0.001	Increase

a) after logarithmic transformation of the taxes; b) 95%CI: 95% confidence interval; c) APC: annual percentage change; d) Prais-Winsten linear regression T-test, 5% significance level.

Individual preventive measures showed a decreasing trend in Brazil (VPA= -6.2; 95%CI -7.7; -4.8), in the Northeast (VPA= -11.9; 95%CI -18.5;-4,8), Southeast (VPA= -3.9; 95%CI -5.5;-2.3) and South (VPA= -7.4; 95%CI -9.9;-5.0) regions; and stability in the other regions.

Moreover, the highest and lowest average rate of individual preventive procedures per 100,000 inhabitants in the 11 years selected, were recorded in the South (17.5 thousand) and Northeast (10.7 thousand) regions, respectively. The overall average of individual preventive procedures in Brazil was 12,400 per 100,000 inhabitants. (Table 1).

It could be seen oscillations in the average rates of dental restoration procedures in the North and Midwest regions between 2008 and 2018 (Figure 2). Brazil (VPA= -7.3; 95%CI -10.5;-3.9) and the Northeast (VPA= -11.0; 95%CI -16.5;-5.1), Southeast (VPA= -5.8; 95%CI -7.4;-4.1) and South (VPA= -3.9; 95%CI -6.3;-1.4) regions showed a decreasing temporal trend. The average rate of dental restoration procedures was 16,600 per 100,000 inhabitants in Brazil, while a higher level was found in the Midwest region (22,700 per 100,000 inhabitants) and a lower level in the Southeast (15,700 per 100,000 inhabitants).

The category of endodontic procedures presented the second lowest average rate of dental procedures in the historical series. In 2010, the Northeast and North regions showed the highest rates (Figure 2) in this group of procedures. The North region showed a decreasing trend in the performance of these procedures (VPA= -9.6; 95%CI -14.6;-4.2), while in the other regions and the country, a stationary trend was found (Table 1).

The average rates of tooth extraction procedures showed a decreasing trend in Brazil (VPA= -6.9; 95%CI -10.5;-3.1), the Southeast (VPA= -7.1; 95%CI -10.8;-3.4) and Midwest (VPA= -6.0; 95%CI -8.3;-3.6) regions, while remained stationary in the other regions (Table 1). The highest oscillations in the average rates of this group of dental procedures were found in the North and Northeast regions. The average rates in the Northeast region were higher than the national ones, throughout the historical series (Figure 2), and obtained the highest average rate (12,700 procedures per 100,000 inhabitants) in the 11 years of the study.

In the group of periodontal procedures, only the Midwest region showed a decreasing trend (VPA= -6.7; 95%CI -10.8;-2.4); in the other regions and nationwide, rates remained stable (p>0.05) (Table 1).

The category of prosthetic procedures showed the lowest average rates. From 2008 to 2018, Brazil performed, on average, 231.6 prosthetic procedures per 100,000 inhabitants. This was the only group of dental procedures to present an upward trend in all regions and in Brazil (Table 1). The highest annual percentage change was observed in the Northeast region, with an increase of 22.3% (95%CI 12.8;32.5).

## DISCUSSION

The analysis showed an overall decreasing trend, as well as in several groups of dental procedures in Brazil, within the scope of the SUS, between 2008 to 2018. With the exception of prosthetic procedures, which showed an upward trend in the country and all its macro-regions, there was a significant decreasing trend in the performance of groups of dental procedures regarding collective and individual preventive measures, dental restorations and tooth extractions. This decreasing trend was even higher in the North and Northeast regions.

In 2008, the sum of collective and individual preventive measures was close to 70% in each region. In 2018, in addition to quantitative reduction of these measures, there was a proportional reduction in these two groups of procedures, indicating that dentists began to devote more time to curative procedures than to collective and preventive measures, when compared to 2008. Changes in both groups of procedures in Brazil, although they showed contrary trends, were found in two previous studies, according to which there was an increase in this production between 1994 and 2007,^13^ and a decrease between 1999 and 2017.^14^

The Northeast region, which concentrated the highest rates of annual procedures in 2008, presented a reduction in these services in 2018, while the South region showed the highest production among the regions in 2018, but with very low production when compared to the early years of the series. Neves et al.^18^ observed important differences among the regions related to the performance of curative procedures, in which the South and Southeast regions showed the highest prevalence, while the North and Northeast showed the lowest prevalence. In the aforementioned study, the highest prevalence of curative procedures was associated with the presence of dental materials and instruments, initial care, continuity of care, home visit performed by the dentist and oral health team modality II. Despite being present and active, the teams did not have physical or material conditions to perform some procedures.

The highest rates of collective and individual preventive procedures were recorded in 2010, taking into consideration Brazil as a unit. When assessing the evolution of supervised toothbrushing (collective procedure) in the historical series from 2008 to 2017, Chaves et al.^19^ pointed out that 2010 was the year with the highest number of these actions in the country, a fact related to the distribution of 40.6 million oral care kits by the Ministry of Health in 4,597 municipalities, in 2009.

The Northeast region stood out for presenting, in the first year of the series, the highest rate of collective and individual preventive measures, and in the last year, the lowest rate related to the same procedures. In addition, the sum of collective and individual preventive procedures fell to less than 50% of total production in this region. This finding corroborates a study by Silva et al.^20^, which observed that, in the 2015-2017 biennium, the outpatient care production of curative procedures was higher than that of preventive procedures in the Northeast region.

In this study, between 2008 and 2018, a significant decreasing trend in the rates of periodontal procedures in the Midwest and a stationary trend in the other regions was found. In the study about the years 1999 to 2017, periodontal procedures showed a linear upward trend in all Brazilian regions,^14^ possibly highlighted by the increase in the record of this group of procedures between 2008 and 2012, while in the early years of the series (1999 to 2007), there were few records related to them.

Other findings of this study differed from those of a study by Chisini et al.,^14^ given the difference between the interval of the years analyzed. The fact that the authors of the aforementioned study had taken into consideration the period before the inclusion of oral health teams in the FHS, their inclusion as of 2002 and the significant increase of these teams and DSCs, provided by the PNSB as of 2004, resulted in an upward outcome for some dental procedures, that had not been verified here.

With regard to this study and its historical series, the highest average rates of tooth extraction and endodontic procedures were related to the Northeast and North regions, and the highest rates of dental restoration procedures, to the Midwest region. The results of the epidemiological survey, SB Brazil 2010, showed that the Midwest, North and Northeast were the regions with the largest number of individuals in need of tooth extraction, endodontic and dental restoration treatments,^8^ a fact that coincides with the procedures provided by the SUS.

The decrease in oral health production in Brazil was followed by a reduction in federal investments in primary health care at the beginning of 2013, its stabilization between 2013 and 2016, and a further reduction in 2017.^19^^).(^^21^ In addition, the Constitutional Amendment, No. 55 of December 15, 2016, was promulgated, and it is responsible for freezing social spending for 20 years, legitimizing new fiscal austerity measures and opening an institutionalized path to reduce investments in public health, and as a consequence, a negative impact on oral health policies.^21^^).(^^22^

Another factor related to the period analyzed are the successive changes in the management of the Ministry of Health and the General Coordination for Oral Health that, since 2015, have presented political instability in the country and generated consequences in the implementation of the Smiling Brazil Program (PNSB).^19^^).(^^22^

The e-SUS AB, an information system implemented by the Department of Primary Health Care of the Ministry of Health since 2013, could have leveraged the record of procedures in the country. However, the new system has not showed positive variations in the rates of dental consultations and procedures recorded on the SIA/SUS.^23^ Although there are few studies on the interference of e-SUS in data recording, studies have pointed to difficulties in the use and feeding of health information through this system in the daily work of health professionals,^24^^).(^^25^ which contributes to incorrect record and underreporting.^26^ Thus, we should reflect on the potential contribution of e-SUS to the improvement of the notification of health actions.

If the inclusion of the e-SUS system is considered as one of the probable causes of the reduction of oral health productivity indicators in the country, the reduction in the federal transfer and difficulties in co-financing at local level, due to the decrease in tax collection and municipal burden in public health funding can also have affected these indicators.^19^ Chaves et al. reaffirm that the causes for this variation need to be investigated through studies on analysis of the implementation and results of oral health policy at regional and municipal levels.

The increase observed in the proportion of prosthetic procedures in all regions of the country should be seen with caution, since this still represents a very low percentage compared to the other categories of procedures. The highest rates of prosthetic procedures were recorded in the Northeast region, a situation also found by Chisini et al.^14^ between 1999 and 2017, resulting both from investment in regional dental prosthetic laboratories and the fact that the region has the largest SUS-dependent population.^14^ The study by Aguiar & Celeste^27^ showed a contradiction between the highest prevalence of edentulous being concentrated in the Southeast region and the largest number of regional dental prosthetic laboratories per inhabitant located in the Northeast region. The regions with the greatest access to these services (except for fixed dental prosthesis) were those with greater availability of the aforementioned laboratories.^27^

The high pent-up demand for prosthetic procedures and population ageing are factors to be taken into consideration, aiming at increasing investment in oral prosthetic rehabilitation. Data from SB Brazil 2010 showed that 92.7% of the elderly and 68.8% of adults needed some type of dental prosthesis.^8^ The inclusion of oral prosthetic rehabilitation in primary health care (in family health centers) and secondary health care (in DSCs), the implementation of regional dental prosthetic laboratories since 2005 and the hiring of private laboratories have been fundamental factors for the upward trend of this group of procedures.^4^ Between 2011 and 2014, there was an increase in the identification of people who needed dental prosthesis, by oral health teams, and as a consequence the increase in prosthetic procedures; however, the frequency of dental prosthesis supply is still considered very low in Brazil.^28^ Between 2003 and 2014, there was an increase in the financial transfer to specialized healthcare. However, between 2015 and 2017, there was stability in the amount allocated to this level of care.^21^

The increase in the total number of oral health teams in Brazil, between 2003 and 2017,^19^ is contrary to the findings of this study that showed a decrease in the rates of dental procedures between 2008 and 2018, suggesting possible limitations related to planning, implementation, follow-up and management of the work of these teams.

However, the growing number of FHS teams, with a small reduction only in 2016, was not reflected in the expansion of population coverage,^19^ therefore further studies are needed.

The limitations of this study, inherent to research using secondary data, refer to the quality and coverage of the records in the information systems, susceptible to errors and underreporting. As positive points, its comprehensive character, the use of data from the Ministry of Health and the possibility of replication of the methodology used in other geographical areas and periods, stood out. Furthermore, the analysis of the categories of dental procedures reflects in the organization, provision and qualification of dental care.

Taking these results, it can be concluded that the dental outpatient production at SUS showed a significant decrease in Brazil between 2008 and 2018, mainly after 2015, with variations among regions and groups of procedures. Only prosthetic procedures showed an upward trend in the period analyzed, either in Brazil or in its macro-regions separately. The results of this study help to understand the evolution of outpatient production in the country and thus can contribute to the management of oral health policies. It can be seen the need to improve planning and permanent monitoring of actions, in addition to greater investment in oral health, so that more Brazilians can have access to health actions and services.
